# The mechanisms of a bifunctional fluorescent probe for detecting fluoride and sulfite based on excited-state intramolecular proton transfer and intramolecular charge transfer

**DOI:** 10.1063/4.0000095

**Published:** 2021-05-27

**Authors:** Xueli Jia, Yonggang Yang, Hongsheng Zhai, Qingqing Zhang, Yuanyuan He, Yang Liu, Yufang Liu

**Affiliations:** Henan Key Laboratory of Infrared Materials & Spectrum Measures and Applications, School of Physics, Henan Normal University, Xinxiang 453007, China

## Abstract

The mechanisms of 2-(Benzo[d]thiazol-2-yl)phenol-based bifunctional probe (HBT-FS) for detecting fluoride (F^−^) and sulfite (SO_3_^2–^) based on excited-state intramolecular proton transfer (ESIPT) and intramolecular charge transfer (ICT) have been theoretically studied. Laplacian bond order of HBT-FS indicates that the F^−^ ion cleaves the Si-O bond and then forms Compound 2 possessing a six-membered ring with a hydrogen bond. Potential energy curves and dynamic simulations confirm that ESIPT in Compound 2 occurs along with this hydrogen bond and forms a keto structure with an emission at 623 nm, which agrees with the observed experimental value (634 nm) after adding F^−^. Therefore, the fluorescence red-shift (from 498  to 634 nm) of HBT-FS observed in experiment after adding F^−^ is caused by ESIPT. The SO_3_^2–^ ion is added to the C_5_ site of HBT-FS, which is confirmed by orbital-weighted dual descriptor, and then forms Compound 3 with fluorescence located at 404 nm. The experimentally measured fluorescence at 371 nm after adding SO_3_^2–^ is assigned to Compound 3. Charge transfer analyses indicate that the ICT extent of Compound 3 is relatively weak compared with that of HBT-FS because of the destruction of the conjugated structure by the addition reaction of SO_3_^2–^, which induces the blue-shift of the fluorescence of HBT-FS from 498 to 371 nm. The different fluorescence responses make HBT-FS a fluorescent probe to discriminatorily detect F^−^ and SO_3_^2–^.

## I. INTRODUCTION

Fluorescent probes have received increasing attention due to their high sensitivity, good selectivity, reliability, noninvasive, and real time detection.^1–6^ A variety of fluorescent probes have been developed to detect cations, anions, and biomolecules *in vitro* and *in vivo*. The different detection mechanisms have been revealed and the common detection strategy is to observe the changes in fluorescence intensity or fluorescence wavelength.^7–16^ Therefore, it is key to design a probe with the fluorescence signal showing obvious change after adding the analyte. Organic molecules with excited-state intramolecular proton transfer (ESIPT) process often exhibit different photophysical properties before and after proton transfer because ESIPT induces a tautomerization process from enol to keto form. In this tautomerization process, the change of geometric structure from enol to keto form induced by the transfer of protons along with hydrogen bonds usually causes the large Stokes shift in fluorescent, which has been excessively studied.^17–28^ This unique photophysical property makes compounds with ESIPT ideal materials for the design of fluorescent probes with many potential applications, such as luminescent materials, photostabilizers, laser dyes, and so on.^29–36^

As one of the benzothiazole derivatives, 2-(Benzo[d]thiazol-2-yl)phenol (HBT) exhibits a typical ESIPT process and is recognized as an ideal fluorophore to design the ratiometric fluorescent probes. So far, many probes for different analytes have been designed and synthesized based on a core of HBT. Wu *et al.* used HBT as fluorophore to synthesize two ratiometric probes (named HBT-ratio-F1 and HBT-ratio-F2) for detecting fluoride ions.^37^ Wang and his co-workers constructed a novel HBT-based near-infrared fluorescent probe for the detection of bisulfite.^38^ Yang *et al.* developed a rhodol derivative that contains HBT as a ratiometric fluorescent probe for sulfite.^39^ These probes have only one recognition group, so they can detect one kind of analyte at a time. However, there is more than one species in the real sample, so it is necessary to develop a multifunctional probe that can distinguish two or more analytes at the same time. Lately, a HBT-based bifunctional ratiometric fluorescent probe 2–(4-(benzo[d]thiazol-2-yl)-3-((tertbutyldimethylsilyl)oxy)benzylidene)malononitrile (HBT-FS) has been designed and synthesized by Song and his co-workers for the discriminative detection of fluoride (F^−^) and sulfite (SO_3_^2–^).^40^ HBT-FS has two sensing groups: (1) tert-butyldimethylsilyl ether moiety is sensitive to F^−^ and (2) C-C double bond is sensitive to SO_3_^2–^. The spectral responses of HBT-FS to F^−^ and SO_3_^2–^ have been measured in the titration experiments, showing that F^−^ and SO_3_^2–^ induced the red-shift and blue-shift of the fluorescence of HBT-FS, respectively. Song and his co-workers proposed that HBT-FS discriminatorily detect F^−^ and SO_3_^2–^ by the combination of the ESIPT and intramolecular charge transfer (ICT) mechanisms. However, the proposed mechanisms lack theoretical verification. The ESIPT process needs to be further theoretically determined because the unique dual fluorescence feature of ESIPT was not observed in the experiment. In addition, the information of ICT cannot be provided through the experiment and needs to be analyzed by theory.

In this work, the theoretical calculations by density functional theory (DFT) and time-dependent density functional theory (TD-DFT) are conducted to investigate photophysical properties before and after the addition of F^−^ and SO_3_^2–^ and reveal the detection mechanisms of HBT-FS to F^−^ and SO_3_^2–^. Laplacian bond order (LBO) and orbital-weighted dual descriptor isosurface are calculated to determine the reaction sites of F^−^ and SO_3_^2–^. The possible geometric configurations of HBT-FS and the products (Compound 2 and Compound 3) in the ground (S_0_) state and the first excited (S_1_) state have been optimized, and the absorption and emission spectra are simulated based on these optimized structures. Moreover, the scanned potential energy curves and dynamic simulations of Compound 2 are performed to investigate the ESIPT reaction kinetics. The frontier molecular orbitals (FMOs) and the isosurface of C_+_ and C_−_ functions are analyzed to explore the ICT properties.

## II. COMPUTATIONAL DETAILS

In this work, the structures of HBT-FS and its products in the S_0_ and S_1_ states are optimized with the DFT and TDDFT methods, respectively. In the optimization process, the structures are not constrained. The transition state is searched with the transition state theory. Frequency analyses confirm that these optimized configurations have no imaginary mode and the transition state has only one imaginary frequency, which is further ensured by the intrinsic reaction coordinate (IRC) calculation. For the above calculations, after testing a large number of functionals (see Table S1), the mPW1PW91 functional combined with TZVP basis set is adopted because of the consistency with the experimental result.^41,42^ Considering the experimental environment, acetonitrile (ACN) is chosen as the solvent by using the integral equation formalism variant of the polarizable continuum model (IEFPCM).^43,44^ The absorption and emission spectra are calculated based on the optimized structures. All calculations in this work are carried out by Gaussian 16 package and dynamic simulations are performed by using Newton-X interfaced with Gaussian.^45,46^ LBO and orbital-weighted dual descriptor as well as the isosurface of C_+_ and C_−_ functions are analyzed by Multiwfn based on the outputs of Gaussian.^47^

## III. RESULTS AND DISCUSSION

### A. Geometric structures

**FIG. 1.**
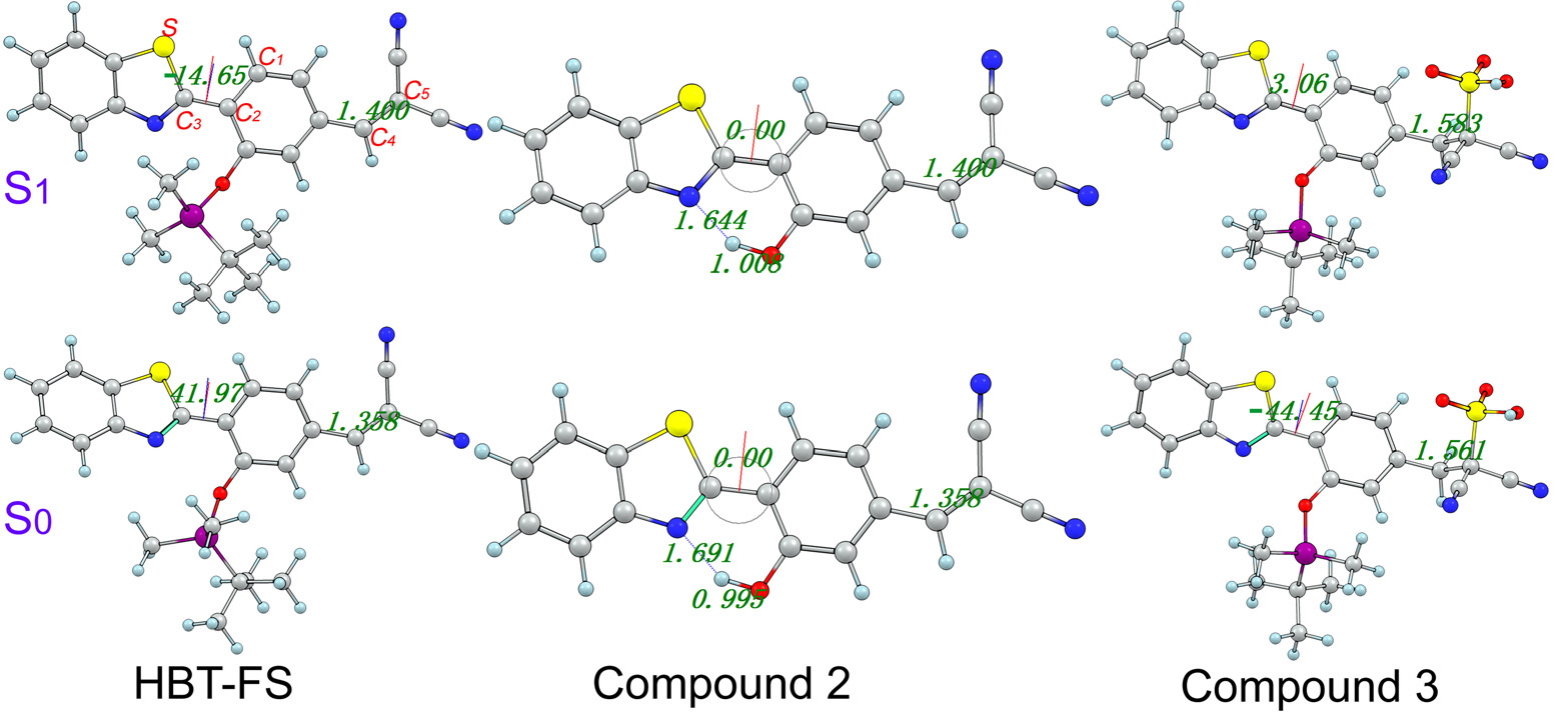
Optimized structures of the fluorescent probe HBT-FS and the corresponding reaction product Compound 2 and Compound 3 at the mPW1PW91/TZVP theoretical level. Blue: N; Light blue: H; Gray: C; Yellow: S; Red: O; Fuchsia: Si.

In order to investigate the sensing progress, the geometries of the fluorescent probe HBT-FS in the S_0_ and S_1_ states have been optimized at the mPW1PW91/TZVP theoretical level. As shown in Fig. 1, HBT-FS is based on a core of 2-(benzo[d]thiazol-2-yl)phenol (HBT) and has two sensing groups, which are the tert-butyldimethylsilyl moiety and the dicyanovinyl group, respectively. The tert-butyldimethylsilyl moiety for detecting F^−^ masks the phenolic hydroxyl group into the ether group, which inhibits the proton transfer process. The dicyanovinyl group for recognizing SO_3_^2–^ is attached to the meta position of the phenolic hydroxyl group in HBT and acted as an electron-withdrawing group. It can be seen from Fig. 1 that the benzothiophene moiety in HBT-FS is not coplanar with the benzene ring, and the dihedral angles δ_(C1-C2-C3-S)_ in the S_0_ and S_1_ states are 41.97° and −14.65°, respectively. The change of the dihedral angle δ_(C1-C2-C3-S)_ from the S_0_ state to the S_1_ state indicates that the benzothiophene moiety is twisted and is more coplanar with the benzene ring in the S_1_ state. The bond length of C_4_-C_5_ double bond in the dicyanovinyl group changes from 1.358 Å in the S_0_ state to 1.400 Å in the S_1_ state, which is extended by 0.042 Å. After adding F^−^, the tert-butyldimethylsilyl ether bond is cleaved to form Compound 2, which is also shown in Fig. 1. In Compound 2, the phenolic hydroxyl group is restored and forms an intramolecular hydrogen bond O-H···N with the adjacent nitrogen atom. The bond lengths of O-H and H···N in the S_0_ state are 0.995 Å and 1.691 Å, respectively. Upon photo-excitation, the bond lengths of O-H and H···N changes to 1.008 and 1.644 Å, respectively. In addition, the bond angle of θ(O-H···N) is increased from 147.6° the S0 state to 148.8° in the S1 state. Based on the lengthening of the O-H bond length and the shortening of the H···N bond length as well as the increase of the bond angle of θ(O-H···N), it can be confirmed that the intramolecular hydrogen bond O-H···N is strengthened in the S_1_ state, which will provide a driving force for proton transfer. The dihedral angles δ_(C1-C2-C3-S)_ in the S_0_ and S_1_ states are both 0°, indicating that Compound 2 has a planar structure. The C_4_–C_5_ double bond lengths of Compound 2 in the S_0_ and S_1_ states are the same as those of HBT-FS, respectively, which are both not changed. For Compound 3 displayed in Fig. 1, it is the product formed after the addition of SO_3_^2–^. The dihedral angle δ_(C1-C2-C3-S)_ in Compound 3 changes from –44.45° in the S_0_ state to 3.06° in the S_1_ state, which demonstrates that the mainframe HBT is more planar in the S_1_ state. However, the C_4_–C_5_ bond length becomes 1.561 Å in the S_0_ state, which is close to the length of C–C single bond. Thus, it can be confirmed that the C_4_–C_5_ double bond of the dicyanovinyl group is broken and that the conjugate system is interrupted by SO_3_^2–^.

### B. Reactive site

**FIG. 2.**
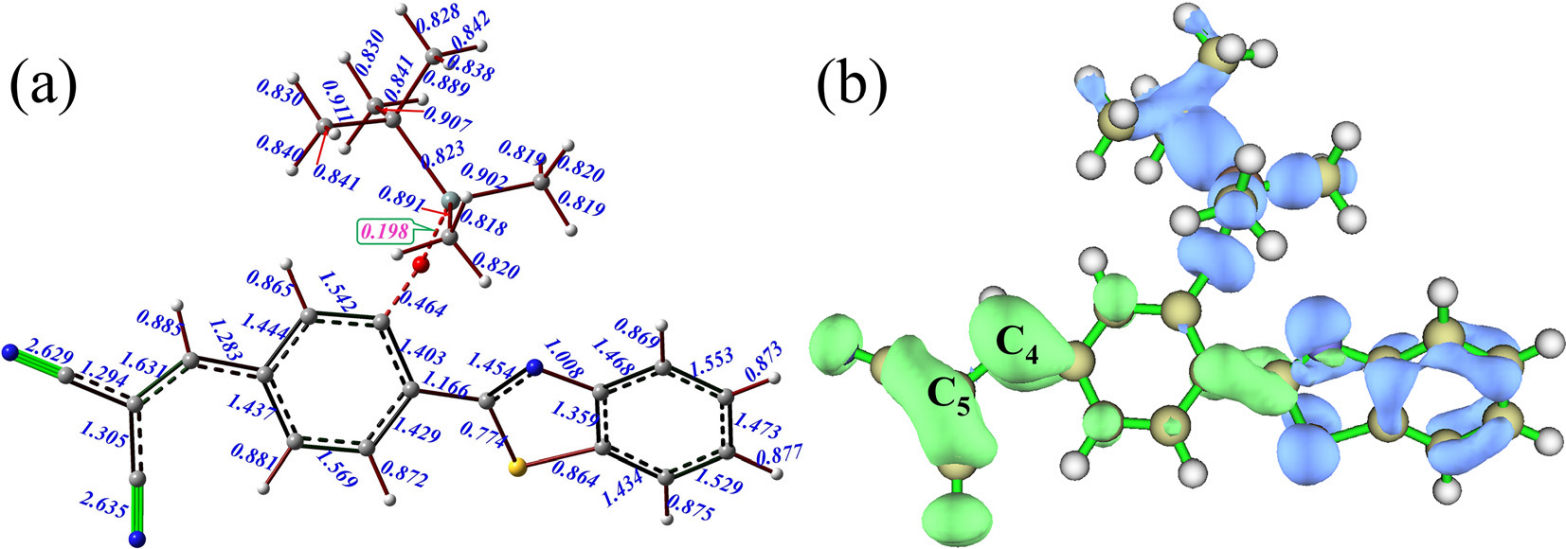
Laplacian bond order (LBO) for all bonds (a) and orbital-weighted dual descriptor isosurface of HBT-FS (b).

Laplacian bond order (LBO) is a newly defined bond order based on a scaled integral of the Laplacian of electron density negative parts in fuzzy overlap space, which can better identify bonding strength because of the direct correlation with the bond dissociation energy.^48^ LBO for all bonds of HBT-FS has been calculated by Multiwfn to determine the cleavage site of F^−^. As shown in Fig. 2(a), the calculated LBO of Si-O bond (0.198) is significantly smaller than that of other bonds, indicating that the bonding strength of Si-O bond is relatively weak. Therefore, the Si-O bond is a favorable cleavage site. In addition, the transition state and the Gibbs free energy barrier of the cleavage reaction are calculated. The searched transition state is verified by IRC (shown in Fig. S1) to be correct. The Gibbs free energy barrier of this reaction is 9.15 kcal/mol. This relatively small energy barrier further indicates that the reaction is prone to occur. For the addition reaction of SO_3_^2–^, the orbital-weighted dual descriptor constructed from Fukui functions is calculated to predict the reactive site.^49^ The green and blue isosurfaces shown in Fig. 2(b) represent nucleophilic and electrophilic regions, respectively. In general, a site with a large green or blue isosurface has remarkable nucleophilicity or electrophilicity. It is noted that the green isosurfaces distributed on the C_4_ and C_5_ atoms of C_4_–C_5_ double bond are relatively large, confirming that the C_4_ and C_5_ atoms are susceptible to undergo nucleophilic attack. In order to quantitatively assess nucleophilicity of the C_4_ and C_5_ sites, the condensed local nucleophilicity indices are calculated based on conceptual density functional, which are 0.0218 and 0.0748, respectively. Therefore, the C_5_ site has high nucleophilicity and SO_3_^2–^ should be added to this site. The transition state is searched and found at the position where the C_4_–C_5_ double bond is broken and the C–S bond is formed, which is further confirmed by IRC calculations (shown in Fig. S2).

### C. Proton transfer in Compound 2

**FIG. 3.**
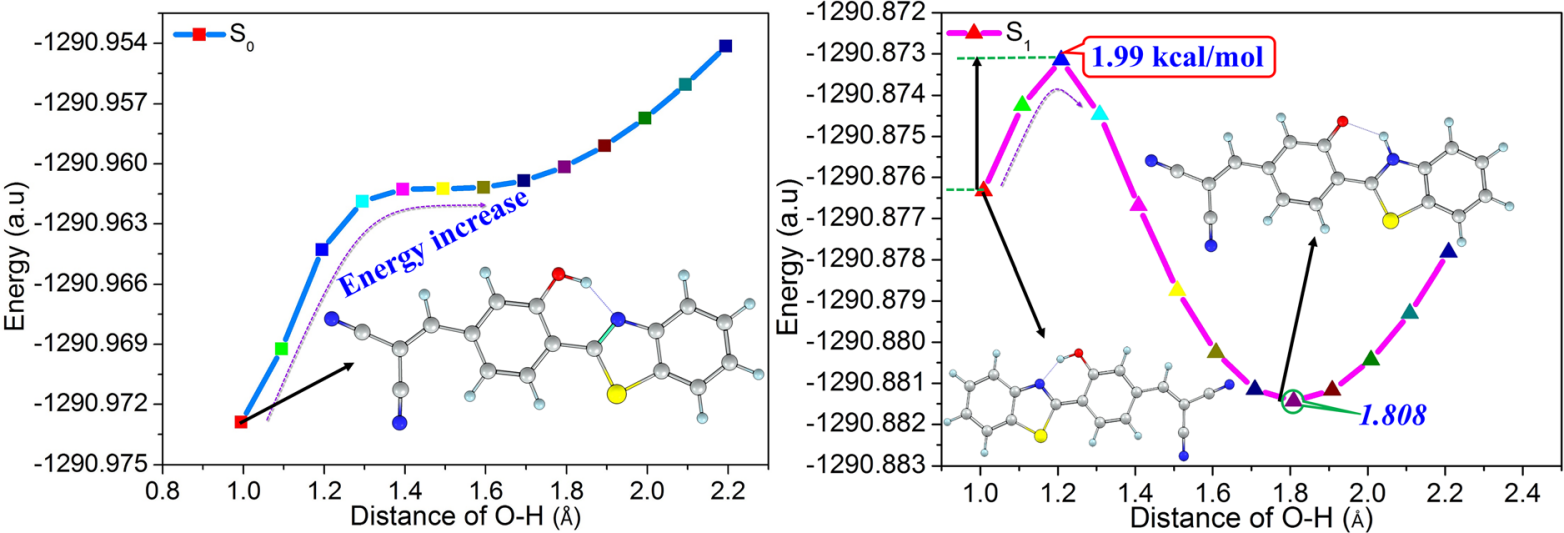
The calculated potential energy curves of Compound 2 in the S_0_ and S_1_ states at the MPW1PW91/TZVP theoretical level.

In Compound 2, the six-membered hydrogen bond ring formed by the phenolic hydroxyl group and the adjacent nitrogen atom provides a possibility for the proton transfer. In order to verify whether proton transfer can occur, the potential energy curves of the S_0_ and S_1_ states are scanned as a function of the O-H bond length, which can provide the change of energy with bond length. As seen from Fig. 3, the energy of the S_0_ state increases with the extension of the O–H bond length, indicating that the proton transfer process in the S_0_ state is unlikely to occur. However, there is an energy barrier of 1.99 kcal/mol between the enol and keto form in the S_1_ state, which is quite small and can be easily overcome. Thus, the proton transfer process of Compound 2 occurs in the S_1_ state and then forms a stable keto structure at the O-H bond length of 1.808 Å.

**FIG. 4.**
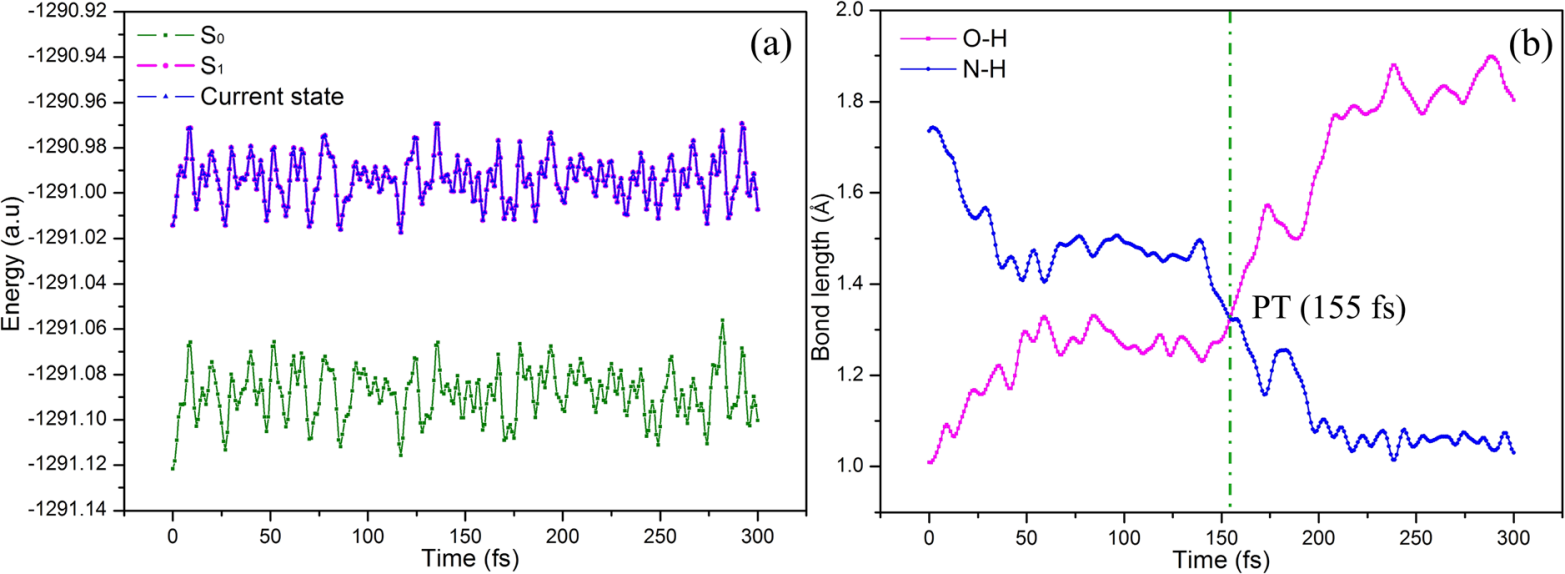
The time evolution of energies (a) and bond lengths (b) in Compound 2.

In order to reveal more details of proton transfer process, the dynamic simulations of Compound 2 are performed by using Newton-X interfaced with Gaussian program. The initial conditions for each trajectory are generated by a sampling procedure using a harmonic-oscillator Wigner distribution for each normal mode. Thirty-two trajectories as a representative set are simulated with a time step of 1 fs under an NVT ensemble at 300 K. The simulated time range is set to 300 fs, which is long enough to cover an ultrafast proton transfer process. In these classical dynamics simulations, results such as state character, energies, and internal coordinates can be described by statistical analysis. The time evolutions of energies and bond lengths are shown in Figs. 4(a) and 4(b). As seen from Fig. 4(a), no surface hopping between the S_0_ and S_1_ states exists, and the current state is located at the S_1_ state, indicating that proton transfer will occur in the S_1_ state. These analyses' results are consistent with that of the potential energy curve analysis. The proton transfer time is defined as the average time in which the distances of the O-H and N-H bonds become equal, that is, the time at the intersection in Fig. 4(b). It can be seen from Fig. 4(b) that the intersection between the two bonds of O-H and N-H indicates proton transfer time constant at 155 fs, which further confirms the occurrence of ESIPT.

### D. Electronic spectra

**FIG. 5.**
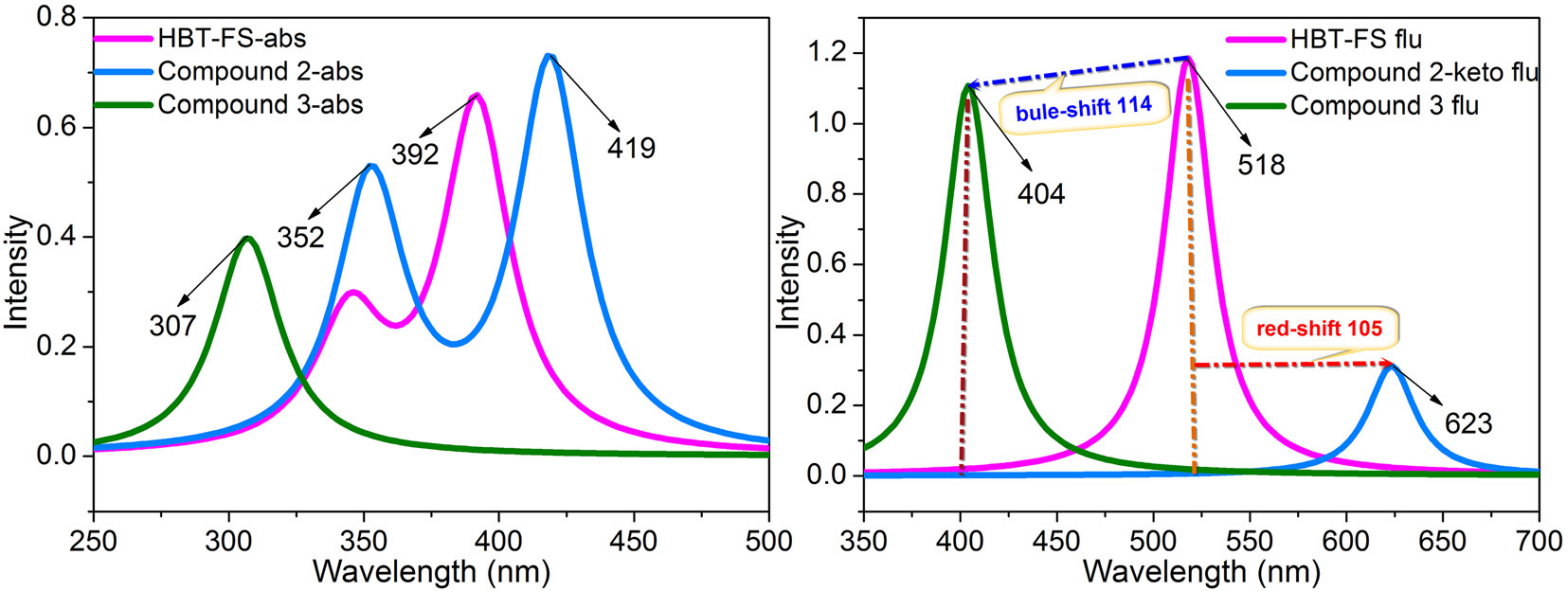
The calculated absorption and fluorescence emission spectra of HBT-FS, Compound 2, and Compound 3 at the mPW1PW91/TZVP theoretical level.

The titration experiment shows that upon the addition of F^−^, the absorption peak at approximately 385 nm decreases gradually accompanying the appearance of two new peaks (approximately 400 and 350 nm). However, after the addition of SO_3_^2–^, only one absorption peak at approximately 300 nm is found. In order to investigate the attribution of these absorption peaks, the six low-lying absorbing transitions of HBT-FS, Compound 2, and Compound 3 are calculated based on the optimized structures of the S_0_ state at the mPW1PW91/TZVP theoretical level. Only the singlet transitions with wavelengths >300 nm are listed in Table I and the calculated absorption spectra are displayed in Fig. 5. As seen from Table I, for HBT-FS, the absorption peak corresponding to the first singlet transition (S_0_→S_1_) is at 392 nm with the oscillator strength of 0.6358, which is in accord with the experimental value (385 nm). For Compound 2, there are two permitted singlet transitions (S_0_→S_1_ and S_0_→S_3_), which are at 419 and 352 nm, respectively. The calculated absorption peaks of Compound 2 coincide with the two peaks (400 and 350 nm) appearing after the addition of F^−^, which further confirms that Compound 2 is the product. The absorption peak of Compound 3 is calculated to be at 307 nm, which is consisted with the experimental data (300 nm). Therefore, the newly formed absorption peak after the addition of SO_3_^2–^ is attributed to Compound 3.

**TABLE I.** The calculated electronic excitation energies (nm), corresponding oscillator strengths, and the corresponding compositions of the low-lying singlet excited states for HBT-FS, Compound 2, and Compound 3.

	Transition	λ (nm)	*f* ^a^	Composition^b^	CI (%)^c^
HBT-FS	S_0_→S_1_	392	0.6358	H→L	95.55%
	S_0_→S_2_	363	0.0066	H-1→L	98.48%
	S_0_→S_3_	345	0.2286	H-2→L	71.09%
	S_0_→S_4_	335	0.0111	H-3→L	73.23%
Compound 2	S_0_→S_1_	419	0.7075	H→L	96.42%
	S_0_→S_2_	359	0.0364	H-1→L	84.99%
	S_0_→S_3_	352	0.4659	H-2→L	81.57%
Compound 3	S_0_→S_1_	307	0.3983	H→L	68.62%

^a^ Oscillator strength.

^b^ H, HOMO (highest occupied molecular orbital) and L, LUMO (lowest unoccupied molecular orbital).

^c^ CI, Composition index.

The fluorescence peak changed with incremental addition of F^−^ and SO_3_^2–^ to the solution of HBT-FS. The fluorescence at 498 nm gradually declined with the increase in F^−^ and a new emission peak at 634 nm appeared simultaneously. For the addition of SO_3_^2–^, the emission peak shifted from 498 to 371 nm. To study the fluorescence properties of HBT-FS, Compound 2, and Compound 3, the emission energies are calculated based on the optimized structures in the S_1_ state. The corresponding calculation results are listed in Table II and the spectral curves are shown in Fig. 5. The calculated emission peak of HBT-FS is at 518 nm, which is in accordance with the experimental value (498 nm). For Compound 2, the S_1_→S_0_ emission energies of the enol and keto structures have been calculated, which are at 511 and 623 nm, respectively. The emission peak of the keto structure agrees with the emission peak at 634 nm appearing after the addition of F^−^. Therefore, the fluorescence peak at 634 nm measured in the experiment is emitted from the keto structure rather than the enol structure, which has a red-shift of 105 nm compared to that of HBT-FS. As for Compound 3, the calculated emission peak is at 404 nm, which is close to the emission peak (371 nm) appearing after the addition of SO_3_^2–^. Thus, the experimentally measured fluorescence of 371 nm is assigned to Compound 3, which has a blue shift of 114 nm compared to that of HBT-FS.

**TABLE II.** The calculated fluorescence emission band in ACN at the mPW1PW91/TZVP theoretical level and the experimental value.

	Form	Transition	λ (nm)	*f*^a^	Composition^b^	CI (%)^c^	Exp. (nm)^d^
HBT-FS		S_1_→S_0_	518	1.1852	L→H	99.07%	498
Compound 2	enol	S_1_→S_0_	511	1.1569	L→H	98.97%	
	keto	S_1_→S_0_	623	0.3117	L→H	99.83%	634
Compound 3		S_1_→S_0_	404	1.1071	L→H	98.51%	371

^a^ Oscillator strength.

^b^ L, LUMO (lowest unoccupied molecular orbital) and H, HOMO (highest occupied molecular orbital).

^c^ CI, Composition index.

^d^ The experimental value of the fluorescence peak.

### E. Charge transfer

**FIG. 6.**
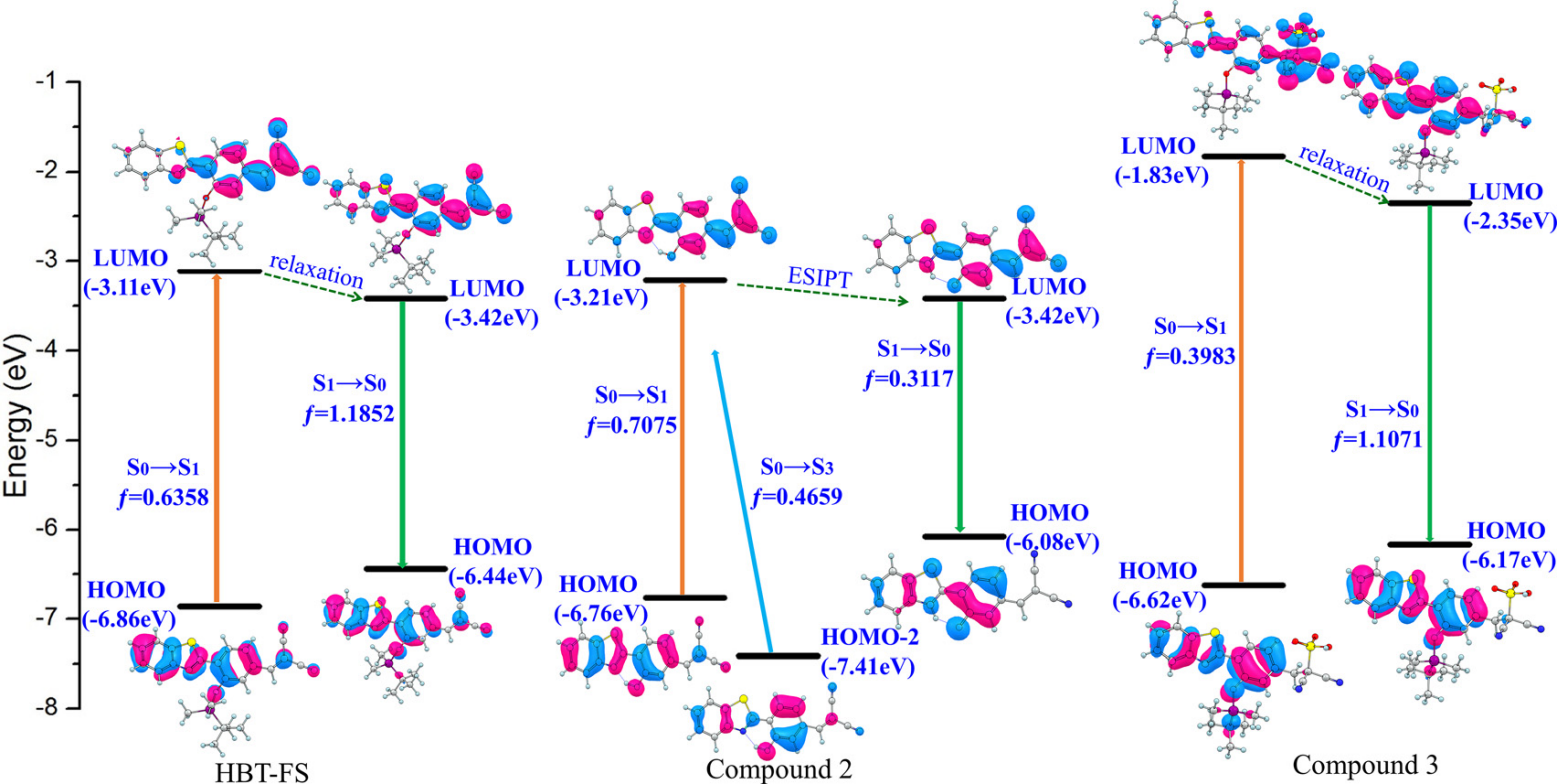
Frontier molecular orbitals involved in the vertical excitation and emission of HBT-FS, Compound 2, and Compound 3.

In order to study the properties of charge transfer and explain the spectral shifts induced by F^−^ and SO_3_^2–^, the frontier molecular orbitals involved in the absorption and emission of HBT-FS, Compound 2, and Compound 3 are shown in Fig. 6. It can be seen from Table I that the first singlet transitions (S_0_→S_1_) for all molecules involve only the electronic transitions from the highest occupied molecular orbital (HOMO) to the lowest unoccupied molecular orbital (LUMO), which are all ππ^*^-type transitions. As seen from Fig. 6, for HOMO of HBT-FS, the electron density is mainly localized on the frame molecule HBT. While for LUMO, it is mainly distributed on the benzene ring and dicyanovinyl moiety. Therefore, charge transfer occurs in HBT-FS upon photo-excitation. For Compound 2, the charge transfer characteristic of the S_0_→S_1_ transition is similar to that of HBT-FS and the second permitted singlet transition (S_0_→S_3_) is dominated by the transition from HOMO-2 to LUMO, which also has a ππ^*^ feature. For Compound 3, the first singlet transition (S_0_→S_1_) corresponding to the transition from HOMO to LUMO involves a charge transition process from HBT to dicyanovinyl moiety. As for the emission of HBT-FS, Compound 2, and Compound 3, the S_1_→S_0_ transitions are all corresponding to the electronic falling from LUMO to HOMO. It can be noted that the electron density on dicyanovinyl moiety in HBT-FS and Compound 2 decreases and ICT in Compound 3 mainly occurs on the frame molecule HBT due to the destruction of the conjugated structure by the addition reaction of SO_3_^2–^. For Compound 2, it can be noted that the energy gap of keto structure (2.66 eV) from LUMO to HOMO is smaller than that of enol structure (3.55 eV), indicating that keto form structure will emit long-wavelength fluorescence. Thus, the red-shift of fluorescence after adding F^−^ is caused by ESIPT. For Compound 3, the energy gap (3.82 eV) from LUMO to HOMO increases compared to that of HBT-FS (3.02 eV), which will emit relatively short-wavelength fluorescence.

**FIG. 7.**
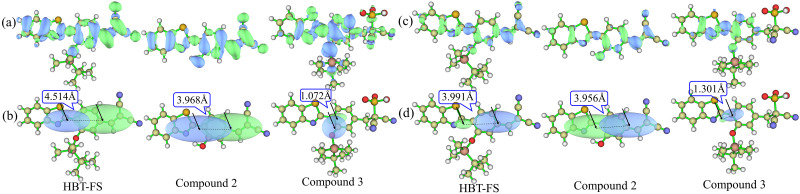
The electron density difference between the S_0_ and S_1_ states in the S_0_ structure (a) and in the S_1_ structure (c). The isosurface of C_+_ (green) and C_−_ (blue) functions as well as the charge-transfer distances for HBT-FS, Compound 2, and Compound 3 in the S_0_ structure (b) and in the S_1_ structure (d).

In order to compare the ICT characteristics between the S_0_ and S_1_ states quantitatively, the electron density differences in the S_0_ and S_1_ structures are calculated for HBT-FS, Compound 2, and Compound 3. Figure 7(a) shows the electron density difference in the S_0_ structure during the electron excitation. The green and blue regions correspond to positive and negative regions, respectively, which represent the increase and decrease in electron density in the electron excitation process. It can be noted that the increase in electron density is mainly in dicyanovinyl moiety, which is consistent with the results of FMOs analysis. In order to visualize the characteristics of charge transfer more intuitively, the isosurfaces C_+_ and C_-_ functions are shown in Fig. 7(b). C_+_ and C_−_ functions proposed by Bahers *et al.* represent two centroids of charges associated with the positive and negative density regions, respectively, which make the direction of electron transfer clearly visible.^50^ In addition, the distance between the barycenters of C_+_ and C_-_ can be used to measure the charge-transfer length to evaluate the charge-transfer degree. In general, the longer the charge-transfer length, the greater the charge-transfer degree. It can be seen from Fig. 7(b) that the charge-transfer lengths between the barycenters of C_+_ and C_−_ in HBT-FS, Compound 2, and Compound 3 are 4.514, 3.968, and 1.072 Å, respectively. Therefore, compared to HBT-FS and Compound 2, Compound 3 has the smaller charge transfer distance, indicating that Compound 3 has a smaller extent of charge transfer characteristic corresponding to the S_0_→S_1_ transition. In addition, the dipole moment variations from the S_0_ state to the S_1_ state for HBT-FS and Compound 2, Compound 3 are 11.30 D, 14.75 D, and 3.19 D, respectively. The insignificant dipole moment variation of Compound 3 further illustrates a weak ICT characteristic in Compound 3.

Figures 7(c) and 7(d) show the electron density differences and the isosurface of C_+_ and C_-_ functions based on the S_1_ structures in the electron de-excitation process, respectively. It can be noted that the charge-transfer lengths for all molecules vary as the structures transfer from the S_0_ state to the S_1_ state. The ICT distances corresponding to the S_1_→S_0_ transition of HBT-FS, Compound 2, and Compound 3 become 3.991, 3.956, and 1.301 Å, respectively. The ICT distance in Compound 3 is still the smaller one compared to that in HBT-FS and Compound 2, confirming a smaller ICT extent. Thus, the addition reaction of SO_3_^2–^ weakens the ICT extent, which leads to the blue-shift (from 498 to 371 nm) of the fluorescence of HBT-FS. In addition, it can be seen that the ICT distances of HBT-FS and Compound 3 change relatively obviously compared to that of Compound 2. This is because the molecular structures of HBT-FS and Compound 3 undergo rearrangement, which has been confirmed by the changes of dihedral angles δ_(C1-C2-C3-S)_ from the S_0_ state to the S_1_ state.

### F. Detection mechanisms

**FIG. 8.**
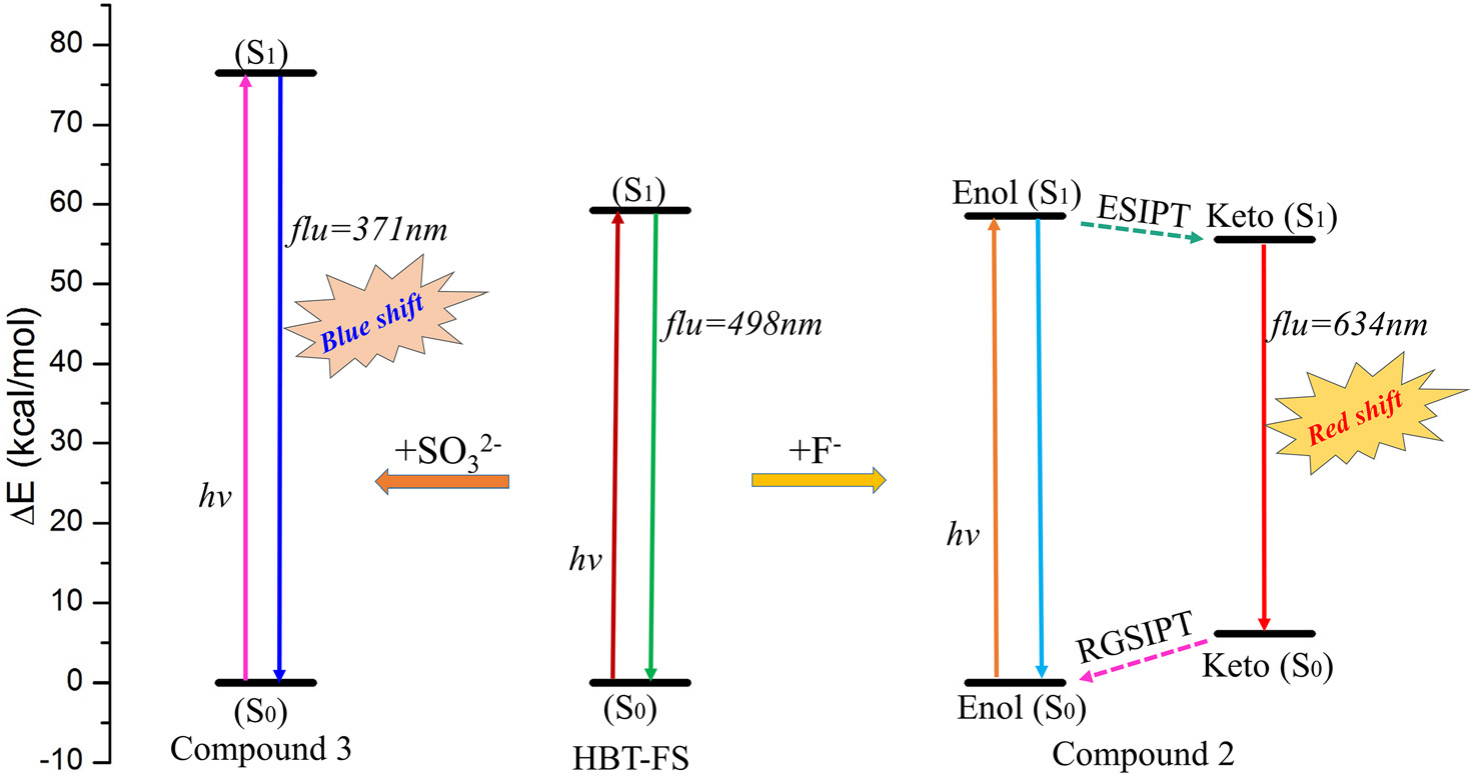
The schematic diagram of fluorescence response mechanisms of HBT-FS for F^−^ and SO_3_^2–^. RGSIPT: Reverse ground state intramolecular proton transfer.

According to the previous spectral measurements and the current theoretical calculations, the fluorescent probe HBT-FS exhibits different fluorescence responses to F^−^ and SO_3_^2–^. Based on the correlation analyses, the detection mechanisms of HBT-FS for F^−^ and SO_3_^2–^ can be plotted as in Fig. 8. HBT-FS with the tert-butyldimethylsilyl moiety and the dicyanovinyl group as sensing groups shows an emission peak at 498 nm. The addition of F^−^ cleaves the tert-butyldimethylsilyl ether bond in HBT-FS to form Compound 2 with a six-membered hydrogen bond ring. Upon photo-excitation, an ESIPT process in Compound 2 occurs along the hydrogen bond to form a keto structure with an emission at 623 nm, which is red-shifted compared to the emission peak of HBT-FS. For the addition of SO_3_^2–^, it will be added to the C_5_ site of the C_4_–C_5_ double bond in HBT-FS to form a Compound 3 with an emission at 371 nm. The addition reaction of SO_3_^2–^ breaks the conjugate structure of HBT-FS and weakens the intramolecular charge transition, thereby inducing the fluorescence blue-shift of HBT-FS from 498 to 371 nm. Based on the different fluorescence behaviors induced by the addition of F^−^ and SO_3_^2–^, HBT-FS can act as a fluorescent probe to discriminatorily detect F^−^ and SO_3_^2–^.

## IV. CONCLUSIONS

In this work, the DFT and TDDFT calculations have been performed to investigate the detection mechanisms of 2-(benzo[d]thiazol-2-yl)phenol-based bifunctional probe (HBT-FS) for F^−^ and SO_3_^2–^. LBO confirms that Si-O bond in HBT-FS is easily cleaved by F^−^ due to a smaller LBO (0.198) and then forms Compound 2 with a six-membered hydrogen bond ring. The constructed potential energy curves and dynamic simulations demonstrate that the ESIPT process in Compound 2 occurs along this hydrogen bond to form a keto structure with an emission at 623 nm, which is consistent with the observed experimental value (634 nm). Therefore, the red-shift of the fluorescence of HBT-FS from 498 to 634 nm after adding F^−^ is caused by ESIPT. For SO_3_^2–^, orbital-weighted dual descriptor isosurface verifies that it is added to the C_5_ site of HBT-FS and then forms Compound 3, which emits fluorescence at 404 nm. Thus, the experimentally measured fluorescence at 371 nm is assigned to Compound 3. In order to explain the blue-shift of the fluorescence of HBT-FS from 498 to 371 nm after adding SO_3_^2–^, ICT is analyzed by frontier molecular orbitals and the isosurface of C_+_ and C_−_ functions. The analysis results demonstrate that ICT extent in Compound 3 is relatively weak compared with that in HBT-FS because of the destruction of the conjugated structure by the addition reaction of SO_3_^2–^, which results in the blue shift of fluorescence of HBT-FS. These different fluorescence responses make HBT-FS a fluorescent probe to discriminatorily detect F^−^ and SO_3_^2–^.

## SUPPLEMENTARY MATERIAL

See the supplementary material for some functionals test results and IRC calculations.

## Data Availability

The data that supports the findings of this study are available within the article.
